# Mental Health, Psychological Features and Psychiatric Comorbidity of Adolescents with Atopic Dermatitis: A Review

**DOI:** 10.3390/pediatric17020050

**Published:** 2025-04-21

**Authors:** Liborija Lugović-Mihić, Dora Bukal, Lorena Dolački, Lucija Zanze, Ema Barac, Renata Tomašević, Maja Vilibić

**Affiliations:** 1 Department of Dermatovenereology, University Hospital Center Sestre Milosrdnice, 10000 Zagreb, Croatia; renatavalo@gmail.com; 2 School of Dental Medicine, University of Zagreb, 10000 Zagreb, Croatia; dora.bukal@gmail.com (D.B.); dolacki.lorena@gmail.com (L.D.); 3 Family Physician Office, 10000 Zagreb, Croatia; lucijazanze15@gmail.com (L.Z.); ema.barac@gmail.com (E.B.); 4 Department of Psychiatry, University Hospital Center Sestre Milosrdnice, 10000 Zagreb, Croatia; maja.vilibic@gmail.com; 5 School of Medicine, Catholic University of Croatia, 10000 Zagreb, Croatia

**Keywords:** atopic dermatitis, mental health, psychological features, psychiatric comorbidity, adolescents, sleep disturbances, depression, anxiety, stress, suicidality

## Abstract

Background/Objectives: Adolescence is a sensitive period of development marked by significant changes. The quality of life (QoL) of adolescents with atopic dermatitis (AD) can be substantially impacted by the disease. The chronic nature of AD is particularly significant: due to recurring (relapsing) skin lesions, adolescents are likely exposed to greater stress and depressive symptoms than those experiencing transient or one-time symptoms. Aesthetic and functional AD skin lesions during adolescence lead to reduced happiness, high stress and depression. Methods: In this review, we wanted to present the current knowledge on mental health, psychological features and psychiatric comorbidity of adolescents with AD, based on the previous studies/research on this topic presented in the PubMed database. Results: Previous studies have confirmed that sleep disturbances, behavioral disorders, internalizing profiles, depression and anxiety, stress symptoms and suicidality represent the most prevalent psychiatric comorbidities and psychological features in adolescents with AD. According to research data, adolescents with AD also reported a tendency toward feelings of sadness and hopelessness, and even suicidal thoughts and attempts. The relationship between sleep disturbances, psychiatric disorders, and suicidality in adolescents with AD is complex and multifaceted. Conclusions: Adequate social competencies are essential for healthy mental development, as their impairments may be associated with psychological alterations or psychiatric disorders in childhood and adolescence that potentially persist into adulthood. These findings highlight the need for continuous psychological evaluation and the implementation of intervention programs from an early age. Psychological interventions, such as cognitive behavioral therapy, accompanied by psychopharmaceuticals, such as selective serotonin reuptake inhibitors (when indicated), seem to be the most beneficial treatment options in AD patients who have the most frequent psychiatric comorbidities: depression and anxiety.

## 1. Introduction

Atopic dermatitis (AD) often poses a significant burden for both sufferers and society due to the chronic and relapsing nature of the disease, which has been increasing in prevalence. According to the research literature, AD is a highly prevalent chronic inflammatory skin condition that affects both adults and adolescents [1]. It impacts approximately 5% to over 20% of children, although prevalence rates vary across countries and regions [2]. Most cases of AD begin before the age of five [2]. Beyond infancy, persistent AD is seen in approximately half of those diagnosed early in life, although AD can also develop in adulthood, accounting for about 25% of adult cases.

The most common manifestations of AD include eczematous skin lesions (acute or chronic), accompanied by itching, which consequently leads to sleep disturbances. Regarding age-specific clinical features of AD, based on Hanifin and Rajka’s criteria, presentation differs between children and adults. For instance, in infants and children, the disease commonly manifests as facial and extensor lesions, while in adults, it is characterized by flexural lichenification or linearity. In addition, concomitant/associated psychological conditions, such as depression, anxiety and sleep disorders, are also frequently observed [3]. Typically, AD runs a chronic and relapsing course. In most children with early-onset AD, the disease resolves by late childhood, though it may persist into adolescence and adulthood [2]. According to one extensive study, 20% of childhood cases had persistent disease eight years post-diagnosis, and up to 5% remained affected 20 years after diagnosis [4]. The age of onset was a key factor associated with AD persistence, as were sex (being female) and severity and duration of the disease.

It is well established that adolescents can also suffer from AD. The number of adolescents diagnosed with AD is gradually increasing, even as many aspects of etiology and treatment approaches remain unclear, classifying AD as a multidisciplinary disease [5,6,7]. Adolescence is a developmental period marked by the transition from childhood to adulthood (typically spanning ages 13–20), during which puberty and significant mental and emotional growth occur. During this phase, autonomy, personality, self-esteem, trust, intellectual abilities and learning capacities develop, which is very important for patients who suffer from AD, because AD can affect self-esteem and other related psychological features.

Although AD primarily manifests on the skin, its impact may extend to other systems, such as the central nervous system and psychological well-being [8]. Depression—either in full syndromal form or, more often, at the subsyndromal level—anxiety and suicidality represent the three most frequent psychiatric comorbidities in patients with AD whether they are children, adolescents or adults [9,10,11]. Adolescence is a particularly sensitive period of development, marked by numerous changes, which warrants special attention to this age group. The quality of life (QoL) of adolescents is significantly affected by the disease, depending not only on disease severity but also on other factors. The co-occurrence of psychiatric symptoms or disorders is the most significant comorbidity—among various other comorbidities—which is an additional factor associated with decreased QoL in adolescents with AD. By nature, AD is a chronic disease, and it has garnered increasing attention in recent decades. However, the burden of AD extends beyond physical symptoms, encompassing psychosocial effects on the individual [12,13,14,15]. Suicide is an issue particular to this sensitive age group regardless—teens face various challenging tasks in this formative period while still having an insufficiently stable personality—while in adolescents with AD, the risk of suicidal ideation, plans or even attempts is increased. Therefore, it is essential to consider the clinical, economic and human burden of AD in both adults and adolescents [3].

## 2. Materials and Methods

In this review we wanted to present current knowledge on the mental health, psychological features and psychiatric comorbidity of adolescents with AD, obtained/based on the previous studies on this topic. Thus, we analyzed data from studies found in the PubMed medical database. The search was performed in December 2024, and it yielded 20 research studies (Table 1) [16,17,18,19,20,21,22,23,24,25,26,27,28,29,30,31,32,33,34,35,36]. To identify relevant publications, we conducted a systematic search for English-language articles featuring the keywords “atopic dermatitis” or “eczema”, “adolescent”, and “mental health” within their titles or abstracts. The search was further refined by applying publication year filters (2000–2024) and age-specific filters, targeting studies involving children (birth–18 years) and adolescents (13–18 years). Subsequently, we manually reviewed the references of selected articles to identify additional studies not captured through the electronic search. To ensure the focus remained on adolescent populations, we excluded articles exclusively addressing children without adolescent data. Furthermore, non-research articles were removed from the final article list. 

## 3. Results

According to the obtained results, there are various studies from all over the world that have been conducted on adolescent patients with AD, and which have analyzed mental health, psychological features and psychiatric comorbidity (Table 1) [16,17,18,19,20,21,22,23,24,25,26,27,28,29,30,31,32,33,34,35,36].

## 4. Discussion

### 4.1. Sleep Disturbances, Internalizing Profiles, Depression and Anxiety, Stress and Suicidality as the Most Prominent Psychological Traits and Psychiatric Comorbidities of Adolescents with AD

Many dermatological patients also suffered from psychiatric comorbidities [7,37,38,39]. This was even more significant for adolescents who undergo a period of life characterized by rapid changes and personal development. Adolescents face the challenge of adapting to their changing bodies, which can trigger increased stress and a reduced sense of happiness, as repeated and chronic inflammatory skin reactions cause visible and functional changes [38]. When individuals, including adolescents, experience negative social and psychological changes, this can lead to depression and anxiety and/or this can be associated with sleep disturbances, specific behavioral patterns, or in the most serious cases, even with suicidality [39]. Previous studies have confirmed that: sleep disturbances, behavioral disorders, internalizing profiles, depression and anxiety, stress symptoms and suicidality represent the most prevalent psychiatric comorbidities and psychological traits in adolescents with AD.

Adolescents with AD have been found to experience more frequent sleep disturbances (primarily due to associated itching), including shorter sleep duration compared to non-atopic adolescents. Disrupted sleep negatively impacts adolescents’ neurocognitive functions and emotional health, increasing the risk of developing mental disorders. Sleep disturbances in AD are particularly notable in those with moderate-to-severe AD [31]. According to research on children, those with active AD report poorer sleep quality. Children with more severe active disease had worse sleep quality, while even children with mild AD experienced sleep disturbances more frequently [40]. Similarly, studies in adults confirm a higher prevalence of fatigue, insomnia and daytime sleepiness among AD patients [41,42].

A study conducted in Brazil predominantly focused on behavioral disorders in adolescents with AD. It found, in this patient population, a predominance of internalizing profiles with anxiety and depression as two of the most common symptoms within it. Oppositional, aggressive behaviors associated with a less present externalizing profile, seem to be more prevalent in a specific subpopulation of adolescents with AD—those with more pronounced, moderate/severe AD [31].

Depression (either in full syndromal form or, more frequently, at the subsyndromal level) and anxiety present significant psychiatric comorbidities in adolescents with AD, as high rates of depression and anxiety are noted in numerous studies [10,19,29,31,43]. One of those studies particularly stressed that adolescents with AD reported not only a high prevalence of depressive symptoms but also a tendency toward feelings of sadness and hopelessness and even suicidal thoughts and attempts [19].

The relationship between sleep disturbances, psychiatric disorders, and suicidality in adolescents with AD is complex and multifaceted. A study by Brazilian authors found high rates of sleep disturbances (60%) and anxiety/depression (25%) but also social problems (32%) in children and adolescents with AD [44]. One study highlighted not only sleep problems but also emphasized concomitant emotional reactivity (mood changes, feelings of panic, worry and emotional vulnerability), cognitive difficulties (worry, rigidity, obsession) and antisocial behaviors (such as peer violence, social exclusion, isolation, discrimination and stigmatization), as well as an increased risk of anxiety and depression as significant, bidirectionally associated components of psychiatric comorbidity in adolescents with AD [45].

In a Korean study of children and adolescents aged 12–18 years, adolescents with AD perceived themselves as unhappy, stressed, depressed and dissatisfied with their sleep quality when compared with adolescents without AD [25]. High levels of psychological stress (59.1%), depression (27.8%) and even suicidal thoughts (13.9%) were found in this specific population. This was especially true of male adolescents with AD, who experienced more pronounced subjective feelings of dissatisfaction/unhappiness (e.g., due to sleep disturbances), implicating the importance of subjective perception of experienced stress. Several studies have confirmed that patients with AD have a high prevalence of sleep disorders, depression, and anxiety but also reduced QoL in the adult, child and adolescent populations [7,19,31,42,46,47,48].

When considering and analyzing psychological factors in patients with AD, gender differences are also noted. Female adolescents were found to be more than twice as likely to report stress and depressive symptoms compared to male adolescents [7]. This aligns with earlier observations that women with AD more often experience high psychological stress, anxiety and depression than men, possibly due to greater concern for their physical appearance, which can have a stronger psychological impact on them [48]. In addition, female adolescents experience new hormonal changes (e.g., menstruation), and symptoms of AD tend to worsen in the premenstrual or ovulatory phase, suggesting that these physiological hormonal differences between the genders may explain significant variations in stress and depression symptoms [7].

The chronic nature of AD is particularly significant. Adolescents with recurring (relapsing) skin lesions are more likely to experience stress and depressive symptoms than those with transient or one-time symptoms [7]. Regarding psychological stress in AD patients, an earlier study reported that 46% of adolescents with high stress levels, and 21% with moderate stress levels, suffer from AD. However, it is important to recognize that stress can be both a cause and a consequence of AD [39,49]. Thus, managing stress levels in adolescents with AD could be crucial for improving patients’ mental health [7]. Other factors, such as socioeconomic status, also influence the condition of patients with AD. Adolescents with AD from low socio-economic backgrounds are more likely to exhibit high psychological stress and depressive symptoms compared to adolescents from middle and high socio-economic backgrounds. Several studies have reported that low socio-economic status is associated with higher stress levels (and elevated cortisol) and a higher incidence of additional dermatoses [50]. Adolescents from low socio-economic backgrounds are also less aware of AD manifestations and often delay diagnosis or have limited access to medical care (diagnosis and treatment) [29].

The severity of AD has a notable impact on psychological status over time. A 10-year follow-up study involving 11,181 children showed an increase in the percentage of depression over time (6% at age 10, 21.6% at age 18) and more frequent symptoms of depression and an internalizing profile in patients with severe AD [29,31]. However, some factors may influence the assessment of behavioral disorders based on the severity of AD, such as comorbid asthma in those with mild AD. It is important to note that adequate social competence is essential for healthy mental development, as its impairment may be associated with psychological disorders in childhood and adolescence and potentially persist into adulthood [31,51,52].

### 4.2. Functioning and Quality of Life, Behavioral Disorders, Attention Disturbances/ADHD and Suicidal Ideations of Adolescents with Atopic Dermatitis

**Figure 1:**
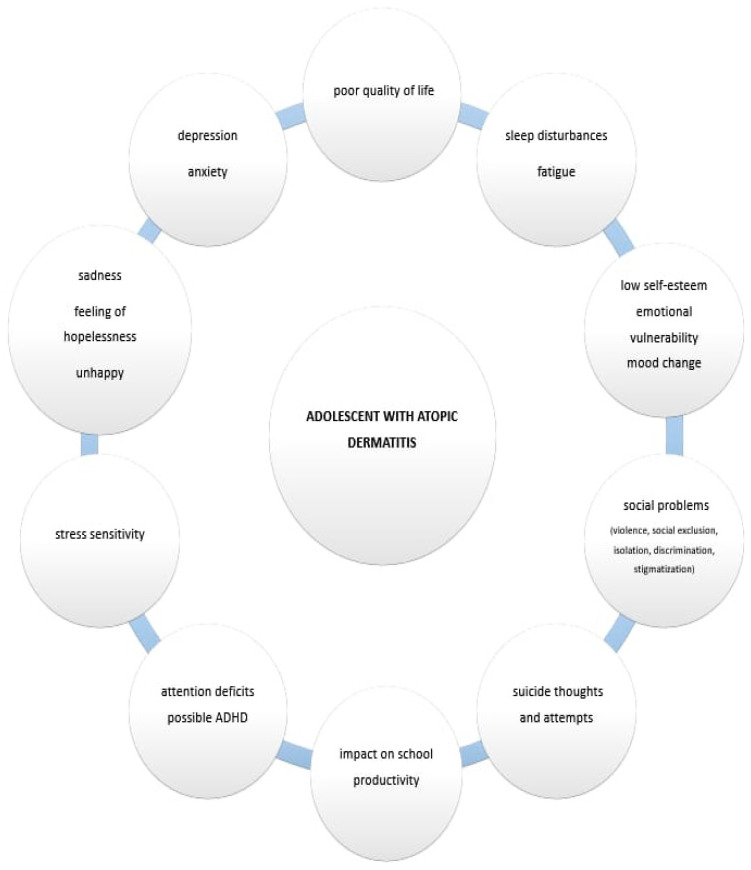
The various aspects of AD in adolescents.

How well an individual functions day-to-day at school/work, socially and with family generally reflects an individual’s well-being. A recent study has documented significant disruptions in AD patients’ QoL such as school or work absences due to AD [53]. Frequent exacerbations and worsening of the disease negatively impact school productivity [54]. Additionally, in one international study, 32% of participants reported that AD affected their school or work life, and 14% of adult participants stated that AD hindered career advancement [54]. Furthermore, AD is strongly associated with more pronounced emotional disturbances, behavioral issues, hyperactivity symptoms and inattention, which often impact social relationships [55]. Adolescents with AD also report greater challenges in peer relationships [27,45]. Figure 1 shows the various aspects of AD in adolescents.

Aesthetic and functional skin lesions due to AD during adolescence lead to reduced happiness, high stress and depression [38]. Previous research has shown that patients/adolescents with AD may struggle with establishing a healthy body image and may experience negative social and psychological consequences [39]. Children and adolescents with AD often exhibit reduced social competence, particularly in activities such as play [31,56]. The previously mentioned study from Brazil revealed impaired social competence in children with AD compared to controls and a significant impact on their daily activities (median: AD 2.5 versus control 5.0). It also reported a higher prevalence of internalizing profiles (median 22.0 versus 12.0), somatic problems (median 5.0 versus 2.0), anxiety/depression (9.0 versus 5.0) and aggressive behavior (18.0 versus 11.0) [31].

Behavioral disorders are relatively common in children and adolescents with AD. One study on 915 adolescents with atopic diseases (mean age 13.3 years) using self-reports and parental assessments found a link between atopic diseases and adolescents’ peer relationship problems [55,57]. Thus, AD was associated with more frequent emotional problems, behavioral issues and hyperactivity/inattention in younger children, likely due to accompanying itching that causes restlessness and inappropriate behaviors. However, AD symptoms often improve during adolescence. In a study of 100 AD patients (mean age 11 ± 3 years), borderline findings or deviations were observed for factors such as overall social competence (75%), internalization (57%), externalization (27%) and aggressive behavior (18%) [31]. Patients with moderate/severe AD had a higher prevalence of aggressive behavior (27.9% versus 10.5%) and sleep disorders (32.6% versus 15.8%) when compared to those with mild AD. Children on immunosuppressive/immunobiological treatments demonstrated lower rates of normal social competence (53% versus 83%). These factors are linked to a higher prevalence of depressive symptoms, stress, suicidal thoughts and suicidal behavior among adolescents with AD [19,24,45]. The severity of AD is important, as moderate/severe cases are more associated with mental disorders (e.g., sleep problems and emotional reactivity) than mild cases, indicating a need for appropriate treatment to address both physical and mental health [44,45].

Attention deficits and possible ADHD are also concerns in these younger patients. Patients with moderate-to-severe AD often experience chronic intense itching and sleep disturbances, which, according to parents and teachers, may lead to attention problems [35]. In one study of 44 participants (mean age 15 years), AD significantly affected sleep, QoL and comorbid anxiety and depression symptoms [35]. Atypical sensory profiles were also reported: sensory hypersensitivity (38.6%), sensory avoidance (50%) and low registration (hyposensitivity, 36.4%) [35]. In adolescents with moderate-to-severe AD without a formal ADHD diagnosis, no significant attention issues were observed compared to similar peers [35]. However, research shows that children with AD report inattention symptoms and a higher prevalence of ADHD-like symptoms. Severe AD in preschool children has been linked to poor sleep and attention dysregulation [58,59,60]. Also, behavioral disorders are prevalent among children and adolescents with AD, primarily internalizing profiles like anxiety and depression [31]. Depression, it should be noted, can lead to suicidal ideation. In a large study involving 72,435 children and adolescents, a higher prevalence of depressive symptoms was observed in AD patients when compared with controls (37.0% versus 30.5%) [19].

Suicidal ideation is particularly pronounced in adolescents. Recent studies have shown a higher prevalence of suicidal thoughts (44%) and suicide attempts (36%) in adolescents with AD when compared to those without AD, highlighting the need for targeted attention around this issue [61]. One study noted that 8% of patients reported suicidal thoughts, unrelated to AD severity [31]. A meta-analysis of six studies on adults and children with AD revealed associations between AD and suicidal ideation, planning (8.0%), and attempts (6.1%) [10].

However, the research published to date shows that most conducted studies are cross-sectional or web surveys. Their results and data lack specific facts, including no clarifications of causal relationships between psychological factors/traits or psychiatric disorders and disease (AD) features. Since most of the studies are cross-sectional, it remains unclear whether AD presents a risk factor for coexisting mental disorders and whether mental disorders and AD share the same underlying pathophysiological mechanisms or if mental disorders exacerbate AD. Thus. further studies are needed for conclusive findings.

### 4.3. Mental Health and Psychological Factors in Relationship to Immune Factors, Neuromediators, the Hypothalamic-Pituitary-Adrenal Axis and Gut–Skin–Brain Interactions in Atopic Dermatitis

According to the research literature, generally, an association between psychological factors and systemic inflammation exists, including the pro-inflammatory cytokines (IL-1, IL-6, and TNF-α). For example, various cytokines and inflammatory mediators play a very important role in depression, a common psychiatric comorbidity in AD patients [62]. Certain features (e.g., fatigue) are characteristics of both disorders—somatic (AD) and psychiatric (depression). The activation of the cytokine network could contribute to the development of depressive disorders/symptoms and sick behavior in patients with AD. This is known as the “cytokine hypothesis of depression”, which emphasizes that depressive symptoms/disorders are regulated by central behavioral and neuroendocrine mechanisms involving neuropathways and various cytokines. According to research data, in patients with somatic diseases (e.g., dermatosis) who have not previously suffered from psychiatric disorders, pro-inflammatory cytokines (including IL-1, IL-6, TNF-α) may cause true major depressive disorders (coexisting with somatic symptoms).

As is known, there is an association between immune factors/the immune system and the CNS, which involves the influence of neuroendocrine factors on immune cells [62,63]. Thus, it needs to be emphasized that AD, ADHD and autism spectrum disorder (ASD) are characterized by similar pathomechanisms, which include inflammation, genetics and changes in the microbiome [64,65]. There are several hypotheses that show similarities/associations between AD and neurodevelopmental disorders. In AD, mast-cell-driven vasoactive mediators might increase blood–brain barrier permeability. Consequently, pro-inflammatory cytokines may pass through the blood–brain barrier, causing potential focal brain inflammation and leading to aberrations in synaptic plasticity. This could subsequently be associated with the occurrence of behavioral conditions like ASD and ADHD [65,66,67]. Thus, neuroinflammation is important for AD because it can modify the metabolism of neurotransmitters. Also, genetic factors, like dysregulated DNA methylation, are very important in both AD and ADHD. In addition, in AD patients, changes in the gut microbiome (gut dysbiosis) may disrupt the gut–brain axis, and could thus contribute to brain dysfunction] [68,69,70].

Also very important is the adequate functioning of the hypothalamic–pituitary–adrenal axis (HPA) axis and the production of hormones that belong to this axis. An increase in stress-triggered endogenous cortisol production predominantly triggers a Th2 cell response, which contributes to allergic conditions such as AD. As is known, cortisol is a crucial stress hormone that may affect cytokines and their activities. For example, cortisol triggers the release of IL-4, which stimulates plasma cells/B cells to produce IgE. In the long term, the effect is that HPA hyperresponsiveness to stress switches to hyporesponsiveness [62]. It is important to mention that hormones that belong to the HPA axis are also usually produced in skin structures (keratinocates, fibroblasts, etc.) and participate in the inflammation of local sites in the skin (peripheral HPA axis). These hormones are related to Th1 i Th2 cells and their products, which may be related to stress duration. Chronic stress leads to a higher Th1 response associated with chronic corticosteroid secretions and IL-10/IL-18 effects, while acute stress causes a changed Th1 response, involving acute corticosteroid secretion and cytokine IL-12/IL-18 effects.

For psychological traits/psychiatric symptoms accompanying AD, it is also important to consider neuroendocrine-mediated communication in the skin–gut axis, which includes the participation of neuropeptides. Thus, psychiatric disorders and psychological traits like depression and anxiety, along with the stress response, may be related to gut microbiota [70]. According to the literature, neuropeptides can stimulate keratinocytes to produce pro-inflammatory cytokines (e.g., IL-1α, IL-4, IL-6, IL-8, IL-10, TNF-α, IFN-γ, etc.) and thus enhance cell migration and antigen expression, influence the presentation of antigens (through Langerhans cells), induce mast cell degranulation with the release of mediators (like histamine) and trigger blood mononuclears to release pro-inflammatory cytokines, which could trigger inflammatory dermatoses like AD [70,71,72]. Concerning psychological/mental stress in relation to the gut, stress can accelerate excessive intestinal microorganism growth, increase intestinal permeability and destroy the intestinal mucosal immune barrier, as well as stimulate the nervous system to release neuropeptides [70]. Also, gut microbiota secretes different metabolites and some of them are hormone-like compounds (like short-chain fatty acids and cortisol) and neurotransmitters (gamma-amino butyric acid, 5-hydroxytryptamine, dopamine and tryptophan), which can penetrate into the blood, after what they act on distant skin [69,70,73].

Finally, according to “the cytokine hypothesis of depression”, in patients/adolescents with comorbid AD and depressive disorder, pro-inflammatory cytokines could trigger behavioral abnormalities, while stimulation of the immune system may be associated with and/or underline other depressive symptoms [62]. Such an observation could have an implication for AD treatment, which is accompanied by the fact that serotonin agonists and selective serotonin reuptake inhibitors (SSRIs) improve the clinical picture of AD and decrease pruritus (although the precise mechanisms are not yet known). It is assumed that the anti-pruritic effect of SSRIs is related to certain mechanisms in the CNS. According to current research results, SSRIs (such as paroxetine, fluoxetine, sertraline, etc.), may improve pruritus, which is a very important therapeutic goal for patients with AD [62].

### 4.4. Coping with Atopic Dermatitis, Positive Measures/Activities and Related Treatment Possibilities

**Figure 2:**
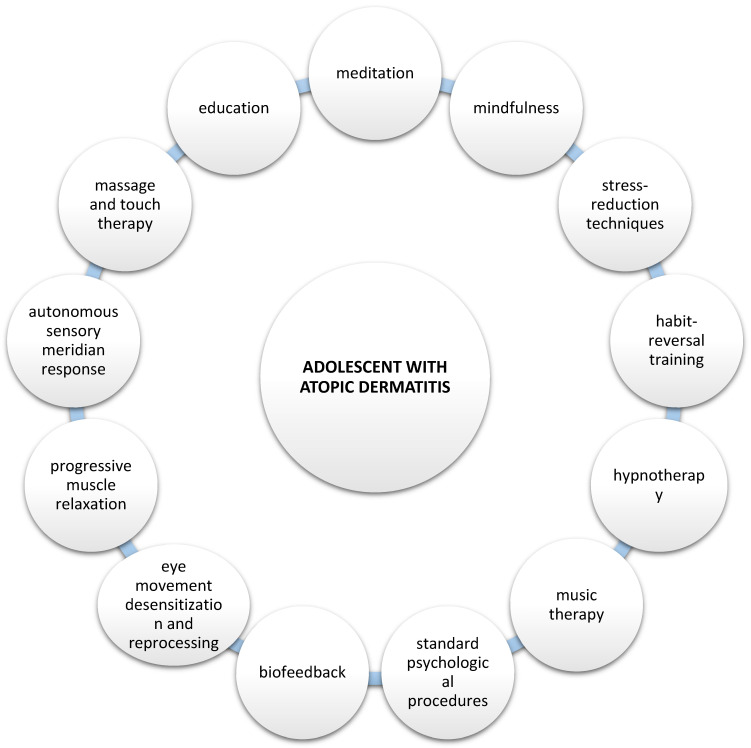
Potential psychological approaches to adolescents who suffer from AD.

According to current global guidelines, the treatment of patients with AD should consider the psychological aspects, which are an important component of the disease and are included in treatment recommendations. In addition to standard dermatological treatment, various approaches have been attempted to help patients. Several additional methods have proven beneficial in more comprehensive treatments of patients, including meditation and mindfulness, stress-reduction techniques, habit-reversal training, hypnotherapy, music therapy, massage therapy and standard psychological procedures such as cognitive behavioral therapy (Figure 2) [74,75,76,77,78,79,80,81,82,83,84].

Continuous psychological observation/follow-up should be conducted, and greater attention should be paid to the psychological changes and/or psychiatric comorbidities, for these patients, especially adolescents with AD [76,77,78]. Medical staff play a crucial role in managing AD patients, including adolescents, through specialized consultations in dermatology clinics. Particular attention should be focused on preventing and promoting mental health in these chronic patients, such as adolescents with AD, recognizing the signs and symptoms of mental disorders, implementing self-help strategies and encouraging healthy lifestyle habits, such as promoting quality sleep and physical activity. In some settings, special programs, including “atopy schools”, are available, which involve education on various aspects of the disease [76,77,78]. Ongoing and continuous support focused on mental health can have a significant impact on adolescents with AD and should be implemented from an early age. The goal of such beneficial intervention is to help adolescents develop skills to cope with the disease and daily stressors, succeed academically, experience satisfaction and feel a sense of community and belonging.

Additionally, an interesting observation from data in the literature indicates that participating in physical activity helps adolescents with AD reduce their psychological stress. Thus, according to other research results, adolescents with AD who are engaged in regular physical activity showed a 30% lower risk of stress compared to those who did not participate [7,79]. This may be because physical activity aids in the reabsorption of cortisol—which is released in significant amounts during a stress response—and helps produce and activate endorphins, which directly affect the brain. Several previous studies have also shown that physical activity contributes to the mental health of patients with allergic diseases such as AD, asthma and allergic rhinitis [80]. However, physical activity may delay the healing of skin lesions in AD patients and accompanying sweat can cause itching, potentially worsening symptoms [81]. Therefore, future studies should provide more information on how to promote physical activity without exacerbating AD symptoms.

Behavioral interventions, particularly cognitive behavioral therapy, seem to be potentially beneficial for patients with AD—with or without a psychiatric comorbidity [82,83,84]. Although most research has included adults with AD, some proposed interventions seem to be suitable for younger age populations, such as adolescents with AD. Cognitive behavioral therapy, either therapist delivered or internet delivered, appears to be efficacious for reducing symptoms of AD as well as accompanying psychiatric symptoms, such as depression or sleep problems [84].

Other beneficial psychological interventions, such as autogenic training and/or biofeedback, could be complementary interventions for adolescents with AD with or without psychiatric comorbidity.

## 5. Conclusions

Overall, these data indicate that in adolescents with AD, the disease negatively impacts their mental health and disrupts their psychological state, thereby affecting their QoL (e.g., via sleep disorders, issues with emotional status and mental health, as well as problems with social functioning, etc.). These findings highlight the need for a psychological approach and the introduction of intervention programs from an early age, such as mental health assessments and professional supervision following diagnosis. Current research results emphasize the importance of a multidisciplinary approach to the comprehensive care of patients with AD, which would include mental health professionals. Promoting support for adolescents with AD is particularly important and should be one of the priorities in the prevention of adverse repercussions of AD on mental health, such as the increased risk of suicide attempts. Finally, appropriate and complementary treatment (dermatological and psychological/psychiatric) of patients with AD, significantly improves their QoL as well as treatment outcomes.

## Figures and Tables

**Table 1:** Data from studies on the mental health, psychological features and psychiatric comorbidity of adolescents with AD.

Author, Year, Study Type	Objectives/Analyzed Factors	Methods and Examinees/Patients	Results	Conclusions
Yaghmaie P, et al., 2013 (cross-sectional study) [16]	To quantify mental health comorbidity in noninstitutionalized children with AD	92,642 children (0–17 years); lifetime prevalence of various mental health conditions, calculated for those with and without a history of AD; data analyzed from the 2007 National Survey of Children’s Health	Odds of having ADHD significantly greater for children with AD. The adjusted odds ratios for depression, anxiety, conduct disorder and autism were 1.81, 1.77, 1.87 and 3.04, respectively	A striking association between mental health disorders and AD in the US pediatric population; a clear dose-dependent relationship between the prevalence of a mental health disorder and reported AD severity
Noh HM, et al., 2016 (web-based survey) [17]	The relationship between suicidal behaviors and AD in Korean adolescents	Prevalence of adolescent health risk behaviors among Korean students from grades 7 to 12; data from the 2012 Eighth Korea Youth Risk Behavior Web-Based Survey (KYRBWS-VIII) (suicidal ideation, planning and suicide attempts, AD, mental health status, etc.)	Significant associations between AD and suicidal behaviors for girls but not for boys. The overestimation of weight perception might have an additive impact on suicidal risk among girls	AD increased the risk of suicidal behaviors in girls in the 7th–12th grades, even after adjustments for multiple confounding factors
Becker-Haimes EM, et al., 2017 (clinical study) [18]	Atopic disease and anxiety as a comorbidity	189 youth who presented for treatment services at a specialty clinic for child/adolescent anxiety and depressive disorders; youth and their parents completed a diagnostic interview and a large number of questionnaires	High rates of atopy in the clinical sample (51.3% reporting history of ≥1 atopic diseases); anxious youth with atopy exhibited more overall and generalized anxiety symptoms relative to non-atopic youth (ps < 0.05)	Comparable or elevated rates of atopy in this clinical sample relative to estimates of community prevalence rates. Youth with anxiety and atopy presented with higher anxiety severity than those without comorbid atopy
Lee S, et al., 2017 (web-based survey) [19]	Association of AD with depressive symptoms and suicidal behaviors among Korean adolescents (2013 Korean Youth Risk Behavior Survey)	72,435 middle and high school adolescents in Korea; self-reporting on AD and yes-or-no answers to questions about depressive symptoms and suicide ideation, suicide planning and suicide attempts	6.8% of adolescents had AD. The proportion reporting depressive feelings was 31.0%, suicide ideation was 16.3%, suicide planning was 5.8% and suicide attempts was 4.2%	Adolescents with AD have a higher prevalence of depression symptoms and suicidal behaviors and are significantly more prone to experience depressive feelings, suicide ideation, suicide planning and suicide attempts than those without AD
Kuniyoshi Y, et al., 2018 (cross-sectional study) [20]	The association between eczema and mental health problems in Japanese school children	9954 participants (2nd to 8th grade) from the 2014 and 2015 ToMMo Child Health Study; ISAAC Eczema Symptom Questionnaire; Clinical ranges of Strengths and Difficulties Questionnaire (SDQ): total difficulties scores and four SDQ subcategories of emotional symptoms, conduct problems, hyperactivity/inattention and peer problems were defined as scores ≥16, ≥5, ≥5, ≥7 and ≥5, respectively	As eczema status worsened, the mean SDQ total difficulties score significantly increased (OR of scores in the clinical range for SDQ total difficulties were 1.51 for mild/moderate eczema and 2.63 for severe eczema, adjusted for sex, school grade, current wheeze, and disaster-related factors, using normal eczema as a reference). The association between eczema severity and the four SDQ subcategories showed a similar trend	A significant association between eczema severity and mental health problems exists. The presence of eczema was associated with four the SDQ subcategories
Kim SY, et al., 2019 (web-based survey) [21]	Exploring psychological distress in Korean adolescents having allergic disease comorbid with obesity	703,869 adolescents completed the Korean Youth Risk Behavior survey between 2007 and 2016; 4 groups (healthy control, allergic disease only, obesity only, and comorbidity of allergic disease and obesity); comparison of mental health between groups	Adolescents with both AD and obesity had significantly greater odds of experiencing unhappiness (OR, 1.17), stress (OR, 1.32), and suicidal ideation (OR, 1.25). The comorbidity groups also showed significantly greater odds of stress and suicidal ideation than the allergic disease-only and obesity-only groups	Allergic disease and obesity negatively and additively influence mental health in adolescents
Hsu DY, et al., 2019 (clinical study) [22]	Association between AD and hospitalization for mental health (MH) disorders in the United States	835 AD (1.36%) and 2,434,703 non-AD (0.75%) patients with primary admission for an MH disorder; data analysis from the Nationwide Inpatient Sample from 2002 to 2012 (containing a representative 20% sample of US hospitalizations)	AD patients admitted for MH disorders are younger, Asian, Black, in a higher income quartile and have an increasing number of chronic conditions. AD was associated with a primary admission for MH disorders in adults, including mood disorders, schizophrenia, and developmental disorders. This association was not present in children	AD was associated with higher odds of hospitalization for all MH disorders and substantial excess costs of inpatient care
Wan J, et al., 2020 (cross-sectional study) [23]	Association between pediatric AD and mental health impairment	Children with and without AD were assessed for mental disorder with impairment (MDI) using a validated behavioral screening questionnaire; mental health services utilization was also reported; United States National Health Interview Survey data (2013–2017)	MDI prevalence was 26.7% among children with AD and 17.7% for those without AD, severe MDI being present in 10.9% and 6.2%, respectively). AD was associated with higher odds of MDI, including impairments in conduct, emotions, peer relationships, and attention	AD is associated with clinically significant mental health symptoms, but many affected children may not seek or receive care
Kyung Y, et al., 2020 (cross-sectional study) [24]	Association of AD with suicide risk	788,411 adolescents, Korean Youth Risk Behavior Web-based Survey; survey data obtained from a stratified, multistage, clustered sample; students self-reported AD if they had received a diagnosis of AD by a physician; influencing factors for suicidal behaviors tested by logistic regression models	Reported suicide ideation: 19.0%; suicide attempts: 4.5%. AD patients were more likely to skip breakfast less frequently, to exercise less frequently, to drink less alcohol, and to not be current smokers and were significantly more likely to have negative mental health states	Adolescents with AD had a meaningful prevalence of suicidal behaviors
Kyung Y, et al., 2020 (cross-sectional study) [25]	Identification of the influencing factors for mental health in adolescents with AD	62,276 participants; 13th Korean Youth Risk Behavior Web-based Survey (KYRBS) conducted in 2017—data obtained from a stratified, multistage, clustered sample. Participants responded to the question “have you ever been diagnosed with AD by a doctor?” and several yes/no questions about stress, depressive symptoms and suicidal ideation	Adolescents with AD were significantly more prone to stress (59.1%), depressive symptoms (27.8%) and suicidal ideation (13.9%). Subjective unhappiness was most strongly associated with stress; depression and suicidal ideation were reciprocally key risk factors	AD in adolescents is associated with a higher prevalence of stress, depressive symptoms and suicidal ideation
Hou A, et al., 2021 (cross-sectional study) [26]	Predictors, and age-dependent pattern, of psychologic problems in childhood AD	Data on 228,898 children (2–17 years old) from the 1997–2018 National Health Interview Survey	Children with AD more commonly experienced depression/sadness, had ADD/ADHD, emotional/behavioral difficulties feelings of frequent worry, and autism. Also, psychologic comorbidity was associated with atopic comorbidities, multimorbidity and being white, households with a lower income and educational background, and no insurance coverage	AD is associated with multiple psychologic disorders, particularly among those who are white, have atopic comorbidities and a low household income Psychologic comorbidities increased in an age-dependent pattern, though in a way like children without AD
Keller W, et al., 2021 (clinical study) [27]	Associations between atopic diseases and behavioral difficulties	2701 participants (3–18 years old), data on behavioral difficulties (Strengths and Difficulties Questionnaire—SDQ) and on atopic diseases (participant’s medical history) Two groups: Group I (3- to 10-year-olds), parent-reported SDQ (n = 1764); Group II (11- to 18-year-olds), parent-reported SDQ (n = 937) and self-reported SDQ (n = 915)	In younger children, AD was strongly associated with higher total difficulties scores, more emotional/conduct problems and more symptoms of hyperactivity/inattention	In younger children, AD is associated with internalizing and externalizing problems. Parents of adolescents are more likely to perceive associations between atopic diseases and behavioral difficulties than the adolescents themselves.
Fishbein A.B, et al., 2021 (cross-sectional study) [28]	Prevalence and severity of sleep disturbance in school-aged children with AD	180 parent–child dyads with AD; stratified sampling based on AD severity (POEM: mild [n = 30], moderate [n = 75], severe [n = 75]), age and race; symptoms of sleep and psychologic health assessed using the Patient-Reported Outcome Measurement Information System	In children ages 5 to 17 with AD, sleep disturbance is common (66.9%). Children who reported sleep disturbance had increased odds of sleep-related impairment, depression, fatigue, and anxiety. Predictors of parent proxy-reported sleep disturbance were itch intensity and low income	Sleep disturbance is a common AD symptom that affects about 3 million US children and is associated with neuropsychiatric impairment, including depression, anxiety, and inattention
Kern C, et al., 2021 (longitudinal cohort study) [29]	Association between AD and internalizing behaviors and symptoms of depression at multiple points across childhood and adolescence	11,181 participants; annual prevalence of AD assessed at 11 points from ages 6 months to 18 years; symptoms of depression (measured using the Short Moods and Feelings Questionnaire at 5 points from ages 10 to 18 years) and internalizing behaviors (measured with the Emotional Symptoms subscale of the Strength and Difficulties Questionnaire at 7 points from ages 4 to 16 years	The period prevalence of symptoms of depression ranged from 6.0% to 21.6%; for internalizing behaviors, from 10.4% to 16.0%. Mild to moderate AD was associated with internalizing behaviors as early as 4 years of age. Severe AD was associated with symptoms of depression and internalizing symptoms	Severe AD is associated with symptoms of depression and internalizing behaviors throughout childhood/adolescence. Increased risk of internalizing symptoms even for children with mild AD beginning early in childhood
Cheng BT, et al., 2021 (cross-sectional study) [30]	The prevalence and predictors of social and behavioral symptoms and functional impairment among US children with AD	2553 US children with AD; behavioral and functional issues examined using Columbia Impairment Scale (CIS) scores	Childhood AD was associated with behavioral/functional problems, particularly nervousness, home behavior, staying out of trouble, and relationships with other kids or siblings. Higher CIS scores in children with AD (vs. without AD and with psoriasis)—higher scores notably associated with male sex, older age, lower household income, public insurance and comorbid depression and anxiety	AD is associated with behavioral and functional impairment, similar to psoriasis and other common chronic conditions
Moraes MM, et al., 2024 (cross-sectional study) [31]	Prevalence and pattern of behavioral problems in children and adolescents with AD	100 AD patients (ages 6–17); assessment of competencies and syndrome scale scores of behavioral problems using the Child Behavior Checklist 6-18 (CBCL 6-18) and AD severity using the EASI score	Borderline/abnormal values for the following: total social competence (75% of patients); internalization (57%); externalization (27%) and aggressive behavior (18%) More common aggressive behavior and sleep disorders in patients with moderate/severe AD than in those with mild AD. Children with current/previous use of immunosuppressants/biological tests had a lower frequency of normal social competence	Common behavioral problems among children/adolescents with AD, with a predominance of internalizing profiles, mainly anxiety and depression. In children with moderate/severe AD, higher prevalence of aggressive behaviors and sleep disorders
Sockler PG, et al., 2024 (cohort study) [32]	The effect of AD on cognitive function and psychiatric comorbidities across early childhood and adolescence	14,975 individuals followed since birth in 1991-92; AD was assessed 11 times (6–166 months); general cognition (IQ) was measured at 18, 49, 103 and 186 months of age using 4 scales (GMDS, WPPSI, WISC, WASI); secondary analyses were stratified by the presence or absence of psychiatric or learning disorder	No significant associations were observed between AD status and full-scale IQ scores on the GMDS, WPPSI, WISC and WASI. However, at age 8, among children with active/moderate-severe AD, WISC Performance IQ and verbal IQ were significantly higher than among those with inactive AD	No clinically meaningful associations between AD activity and severity and general cognitive function during early childhood and adolescence
Cai XC, et al., 2024 (analysis of seven electronic databases) [33]	Epidemiology of mental health comorbidity in AD patients	Analysis of 7 electronic databases from creation to October 2022; the Agency for Healthcare Research and Quality (AHRQ) and Newcastle–Ottawa Scale (NOS) tools were used to assess the quality of observational studies	1998 October 2022 global prevalence rates in AD patients: ADHD (7%), depression (17%), anxiety (21%) and suicidal ideation (13%). Among children aged < 18, North American children had highest rates of ADHD (10%), depression (13%) anxiety (20%). Among adults (aged ≥ 18), adults in Africa had highest rates of depression (36%) and anxiety (44%); Adults in Asia had highest rates of ADHD (7%), suicidal ideation (20%)	Results show high prevalence and comorbidity rates of mental illnesses with AD
Paller AS, et al., 2024 (cross-sectional study) [34]	Stigmatization and mental health impact of chronic pediatric skin disorders	1671 children (ages 8–17 years) with chronic dermatoses and 1 parent. The extent of stigma with child-, caregiver- and physician-assessed disease visibility (primary outcome) was compared to (a) severity, using the PROMIS Stigma-Skin, (b) reduced QOL (assessed by Skindex-Teen), (c) depression, (d) anxiety and (e) poor peer relationships (PROMIS child and proxy tools) (secondary outcomes)	56.4% of participants had high disease visibility, 50.5% had moderate disease severity. Stigma scores significantly differed between physician-assessed and child/proxy-assessed disease visibility and severity. Among children with chronic dermatoses, only 27.0% had minimal or no stigma, 43.8% had at least moderate stigma. Stigma scores correlated strongly with reduced QOL, depression, anxiety and poor peer relationships	Physician assessment of disease severity and visibility is insufficient to evaluate disease impact on the patient/caregiver
Paller AS, et al., 2024 (cross-sectional study) [35]	Attentiveness and mental health in adolescents with moderate-to-severe AD without ADHD	44 AD patients (ages 12–17 years) (moderate-to-severe AD) without clinician-diagnosed ADHD; Attention was evaluated using Conners CPT-3 and the Stroop Color and Word Test; lesional severity measures included EASI and BSA involvement; subjects completed self-report rating scales for sensory responsiveness patterns (AASP), itch (PP-NRS), skin pain, QOL, sleep, anxiety and depressive symptoms	Substantial AD impact on sleep, QOL, and comorbid anxiety and depressive symptoms. According to subject-level data review by two psychologists, only 2 subjects demonstrated an overall response pattern that clearly indicated attention deficit	Adolescents with moderate-to-severe AD without an existing ADHD diagnosis did not demonstrate greater attention problems on performance-based measures than would be expected in age/gender-matched peers
Blanco Sequeiros A, et al., 2024 (clinical study) [36]	Psychiatric comorbidities of childhood-onset AD in relation to eczema severity	Children diagnosed with AD before the age of 12; patient health record data obtained from the Finnish Health Register for Health Care (CRHC); diagnosis codes for psychiatric comorbidities following AD diagnosis were searched for in CRHC for individuals aged < 18 and ≤ 30 years	Increased risk of several psychiatric disorders associated with increased severity in childhood-onset AD (not for other examined factors, i.e., depression, anxiety disorders, panic disorder and bipolar disorder)	Childhood-onset AD is associated with different psychiatric comorbidities depending on AD severity

Abbreviations: AASP—Adult/Adolescent Sensory Profile; AD—atopic dermatitis; ADD/ADHD—attention deficit (hyperactivity) disorder; AHRQ—Agency for Healthcare Research and Quality; BSA—body surface area; CBCL 6-18-Child Behavior Checklist 6-18; CI—confidence interval; CIS—Columbia Impairment Scale; CPT-3—Continuous Performance Test, Third Edition; CRHC—Finnish Health Register for Health Care; EASI—Eczema Area Severity Index; GMDS—Griffiths Mental Development Scales; IQ—intelligence quotient; ISAAC—International Study of Asthma and Allergies in Childhood; KYRBS—Korean Youth Risk Behavior Web-based Survey; KYRBWS-VIII—Eighth Korea Youth Risk Behavior Web-Based Survey; MDI—mental disorder with impairment; MH—mental health; n-number; NOS—Newcastle–Ottawa Scale; OR—odds ratio; PP-NRS—Peak Pruritus Numerical Rating Scale; PROMIS—Patient-Reported Outcomes Measurement Instrumentation System; QO—quality of life; SDQ—Strengths and Difficulties Questionnaire; SD—standard deviation; ToMMo—Tohoku Medical Megabank Organization; US—United States; WASI—Wechsler Abbreviated Scale of Intelligence; WISC—Wechsler Intelligence Scale for Children; WPPSI—Wechsler Preschool and Primary Scale of Intelligence.

## Data Availability

The data that support the findings of this study are openly available in PubMed or available in other sources.

## Abbreviations

The following abbreviations are used in this manuscript:

AASP	Adult/Adolescent Sensory Profile;
AD	atopic dermatitis;
ADD/ADHD	attention deficit (hyperactivity) disorder;
AHRQ	Agency for Healthcare Research and Quality;
BSA	body surface area;
CBCL 6-18	Child Behavior Checklist 6-18;
CI	confidence interval;
CIS	Columbia Impairment Scale;
CPT-3	Continuous Performance Test, Third Edition;
CRHC	Finnish Health Register for Health Care;
EASI	Eczema Area Severity Index;
GMDS	Griffiths Mental Development Scales;
IQ	intelligence quotient;
ISAAC	International Study of Asthma and Allergies in Childhood;
KYRBS	Korean Youth Risk Behavior Web-based Survey;
KYRBWS-VIII	Eighth Korea Youth Risk Behavior Web-Based Survey;
MDI	mental disorder with impairment;
MH	mental health;
N	number;
NOS	Newcastle–Ottawa Scale;
OR	odds ratio;
PP-NRS	Peak Pruritus Numerical Rating Scale;
PROMIS	Patient-Reported Outcomes Measurement Instrumentation System;
QOL	quality of life;
SDQ	Strengths and Difficulties Questionnaire;
SD	standard deviation;
ToMMo	Tohoku Medical Megabank Organization;
US	United States;
WASI	Wechsler Abbreviated Scale of Intelligence;
WISC	Wechsler Intelligence Scale for Children;
WPPSI	Wechsler Preschool and Primary Scale of Intelligence.
